# Reproducibility of Quantitative Brain Imaging Using a PET-Only and a Combined PET/MR System

**DOI:** 10.3389/fnins.2017.00396

**Published:** 2017-07-17

**Authors:** Martin L. Lassen, Otto Muzik, Thomas Beyer, Marcus Hacker, Claes Nøhr Ladefoged, Jacobo Cal-González, Wolfgang Wadsak, Ivo Rausch, Oliver Langer, Martin Bauer

**Affiliations:** ^1^Center for Medical Physics and Biomedical Engineering, Medical University of Vienna Vienna, Austria; ^2^Department of Radiology, Detroit Medical Center, Children's Hospital of Michigan, Wayne State University School of Medicine Detroit, MI, United States; ^3^Department of Biomedical Imaging and Image-guided Therapy, Division of Nuclear Medicine, Medical University of Vienna Vienna, Austria; ^4^Department of Clinical Physiology, Nuclear Medicine and PET Rigshospitalet, Copenhagen, Denmark; ^5^CBmed GmbH, Center for Biomarker Research in Medicine Graz, Austria; ^6^Department for Clinical Pharmacology, Medical University of Vienna Vienna, Austria; ^7^Health and Environment Department, AIT Austrian Institute of Technology GmbH Seibersdorf, Austria

**Keywords:** kinetic modeling, PET/MR, PET, attenuation correction, inter-scan variability

## Abstract

The purpose of this study was to test the feasibility of migrating a quantitative brain imaging protocol from a positron emission tomography (PET)-only system to an integrated PET/MR system. Potential differences in both absolute radiotracer concentration as well as in the derived kinetic parameters as a function of PET system choice have been investigated. Five healthy volunteers underwent dynamic (R)-[^11^C]verapamil imaging on the same day using a GE-Advance (PET-only) and a Siemens Biograph mMR system (PET/MR). PET-emission data were reconstructed using a transmission-based attenuation correction (AC) map (PET-only), whereas a standard MR-DIXON as well as a low-dose CT AC map was applied to PET/MR emission data. Kinetic modeling based on arterial blood sampling was performed using a 1-tissue-2-rate constant compartment model, yielding kinetic parameters (K_1_ and k_2_) and distribution volume (V_*T*_). Differences for parametric values obtained in the PET-only and the PET/MR systems were analyzed using a 2-way Analysis of Variance (ANOVA). Comparison of DIXON-based AC (PET/MR) with emission data derived from the PET-only system revealed average inter-system differences of −33 ± 14% (*p* < 0.05) for the K_1_ parameter and −19 ± 9% (*p* < 0.05) for k_2_. Using a CT-based AC for PET/MR resulted in slightly lower systematic differences of −16 ± 18% for K_1_ and −9 ± 10% for k_2_. The average differences in V_*T*_ were −18 ± 10% (*p* < 0.05) for DIXON- and −8 ± 13% for CT-based AC. Significant systematic differences were observed for kinetic parameters derived from emission data obtained from PET/MR and PET-only imaging due to different standard AC methods employed. Therefore, a transfer of imaging protocols from PET-only to PET/MR systems is not straightforward without application of proper correction methods.

**Clinical Trial Registration:** www.clinicaltrialsregister.eu, identifier 2013-001724-19

## Introduction

Over the last decades, positron emission tomography (PET) imaging has proven its value in both neurological research and in the clinical domain, first using PET-only systems and then, more recently, combined PET/CT systems (Bohnen et al., 2012). PET/CT systems are currently the mainstay of molecular imaging departments thanks to the markedly reduced scan time (owing to faster CT-based transmission scans) and the intrinsic alignment of molecular information to a high-resolution anatomical background (Townsend et al., 2004).

The coincidence detection principle of PET imaging allows accurate correction for attenuation (ATN), and thus, absolute quantification of radiotracer concentration in tissue (Kotasidis et al., 2014). Furthermore, PET permits the quantitative assessment of radiotracer exchange rate constants between blood and different tissue compartments based on kinetic modeling (Kotasidis et al., 2014). The exact values for all of these measures, however, depend on system performance parameters, such as spatial resolution, crystal efficiency, or the applied attenuation correction (AC) method, that are likely to differ across the range of PET/CT systems offered by multiple vendors (Moody et al., 2015; Walker and Sossi, 2015).

Given this caveat, change in instrumentation during ongoing projects is usually avoided at all cost in order to prevent an unnecessary increase in data variability. Unfortunately, the PET-only systems that have been used in the past for neuroimaging research near their end-of-life cycle and ongoing research projects need to be migrated to either dual-modality PET/CT or PET/MR systems. As a result, there is a need to better understand how the transfer of imaging protocols will affect quantitative parameters derived from molecular imaging studies and how to minimize their potential impact on longitudinal research projects.

At our center—similar to Nuclear Medicine operations elsewhere—a PET-only system has been used for many years to perform numerous brain imaging studies (Keller et al., 2013). Correction of photon ATN in this PET-only system is based on a measured transmission (Tx) scan that is subsequently converted into an ATN map (Ostertag et al., 1989). Despite the fact that this method is most appropriate for the correction of coincidence photons in tissue, the method suffers from relatively long Tx scan acquisition times (>10 min per bed position) in order to obtain ATN maps with sufficiently low noise level (Holm et al., 1996). In an attempt to decrease Tx scan times, tissue segmentation of short Tx scans (<5 min) has been suggested (Bettinardi et al., 1999). The segmented ATN maps are produced by segmenting the acquired AC maps into two compartments. Following the compartment separation, the original ATN values are re-inserted using different weights obtained, whereby the segmentation of soft and bone tissue is decided automatically. The segmented AC maps do, however, introduce invariant ATN values for the bones, which can affect regional quantifications (Bettinardi et al., 1999; Keller et al., 2013). Thus, even inter-system variations among PET-only systems can yield differences of up to 10%, as demonstrated in a comparison between two PET systems from the same vendor (Siemens): an ECAT HRRT system and an ECT HR+ system (van Velden et al., 2009, 2014). Hence the migration of research protocols between different PET systems is problematic and warrants a detailed assessment of the underlying causes in order to develop strategies to address potential quantitative differences arising from inherent instrumentation.

Since a few years, combined PET/MR systems are being considered as the modality-of-choice for neurological PET examinations (Catana et al., 2012). The advantage of a PET/MR system for brain imaging is its versatility in using various MR sequences that can provide additional information (functional magnetic resonance imaging, diffusion tensor imaging, and magnetic resonance spectroscopy) and that complement and augment functional information derived from PET, as well as a reduce radiation exposure to the patient due to the lack of a CT component. Despite the promises of this novel technology, however, outstanding methodological challenges remain, most prominently the creation of MR-derived ATN maps to correct for photon ATN in brain tissue (Martinez-Moller et al., 2009). Although this is a fast-moving field of innovation, differences have been observed between the standard DIXON-ATN map and a CT-derived ATN map, especially in tissue close to bones (Samarin et al., 2012; Andersen et al., 2014) As a result, the quantitative accuracy of PET-derived regional radiotracer concentration measurements might be compromised and be no longer comparable to PET-based quantification using PET-only or PET/CT systems. Lately, however, new experimental AC methods have been proposed by various academic groups yielding marked improvements in quantitative accuracy, particularly in areas close to bone (Ladefoged et al., 2017). All of these methods all outperform the standard DIXON AC method and warrant further validation and adoption into commercial offerings.

In view of the lack of wider accessibility of these new AC methods and local requirements that may prohibit the implementation of non-certified correction methods on certified PET/MR systems, the consequences of imaging protocol migrations from PET-only systems to state-of-the-art PET/MR systems require further attention. To this end, the objective of our study was to determine whether results of imaging protocols performed with a PET-only system are comparable to results obtained using a state-of-art PET/MR system. More specifically, the PET-only nears its end of lifecycle and soon is to be replaced while various neurological studies involving numerous subjects are still ongoing.

Here we report the findings for one representative radiotracer, (R)-[^11^C]verapamil, which can be used to measure the function of the multidrug efflux transporter P-glycoprotein (P-gp) at the blood-brain barrier (BBB; Lubberink et al., 2007; Bauer et al., 2012; Römermann et al., 2013). We chose this radiotracer for its simplicity, as P-gp function at the BBB can be characterized by a simple 1-tissue 2-rate constant (1T2K) compartment model (Lubberink et al., 2007). Our objective was to determine potential differences in both absolute radiotracer concentration as well as in the derived kinetic parameters as a function of PET system choice.

## Materials and methods

### Subjects

The study was registered with EudraCT (number 2013-001724-19), approved by the Ethics Committee of the Medical University of Vienna and the national competent authority and conducted in accordance with the Declaration of Helsinki and its amendments. All subjects gave written informed consent before entry into the study. Volunteers were confirmed to be healthy based on medical history, physical examination, routine laboratory tests, urine drug screening, electrocardiography, and vital signs and had to be drug free for at least 2 weeks. Two female and three male healthy volunteers (mean age: 25 ± 1 y, mean weight: 71 ± 14 kg) were included in this study.

### Radiotracer synthesis

(R)-[^11^C]verapamil was synthesized following a previously published procedure and formulated in sterile 0.9% (w/v) aqueous saline solution/ethanol (9/1, v/v) (Langer et al., 2007). Radiochemical purity was >98% and specific activity at time of injection was >30 GBq/μmol.

### Imaging and blood processing protocol

Brain imaging studies were performed using both a PET-only (GE Advance) and a PET/MR (Siemens Biograph mMR) system. All subjects were injected intravenously with (R)-[^11^C]verapamil (364 ± 42 MBq) over 20 s in both systems. For PET-only, a dynamic emission sequence was acquired in 3D mode for 40 min, consisting of 18 frames with increasing frame duration (15 s to 10 min). For PET/MR, 3D PET data was also acquired for 40 min in list mode and rebinned to the same frame sequence as used for PET-only. Standard AC was performed based on the DIXON-MR sequence provided by the manufacturer (Martinez-Moller et al., 2009). In addition, a low-dose CT was acquired on a whole-body PET/CT system (Siemens Biograph TPTV, Siemens Medical Solutions, USA) in order to allow for an alternative AC. The low-dose CT was co-registered to the orientation of the DIXON-ATN map, and converted to ATN values using a bi-linear correction factor (Carney et al., 2006).

A balanced study design was used, in which three subjects were imaged in the PET/MR system first, and the remaining two were imaged in the PET-only system first. An interval of 3 h was used between the two imaging protocols in which the subjects underwent a low-dose CT scan for AC purposes (tube voltage = 120 kV, mA = 194, slice thickness = 3 mm). Arterial blood samples were collected from the radial artery for both imaging protocols (PET-only and PET/MR) through an arterial catheter. Selected arterial plasma samples were analyzed for polar radiolabeled metabolites of (R)-[^11^C]verapamil using a solid-phase extraction protocol as described previously (Langer et al., 2007).

### Attenuation correction, data processing, and pet image reconstruction

**Figure 1:**
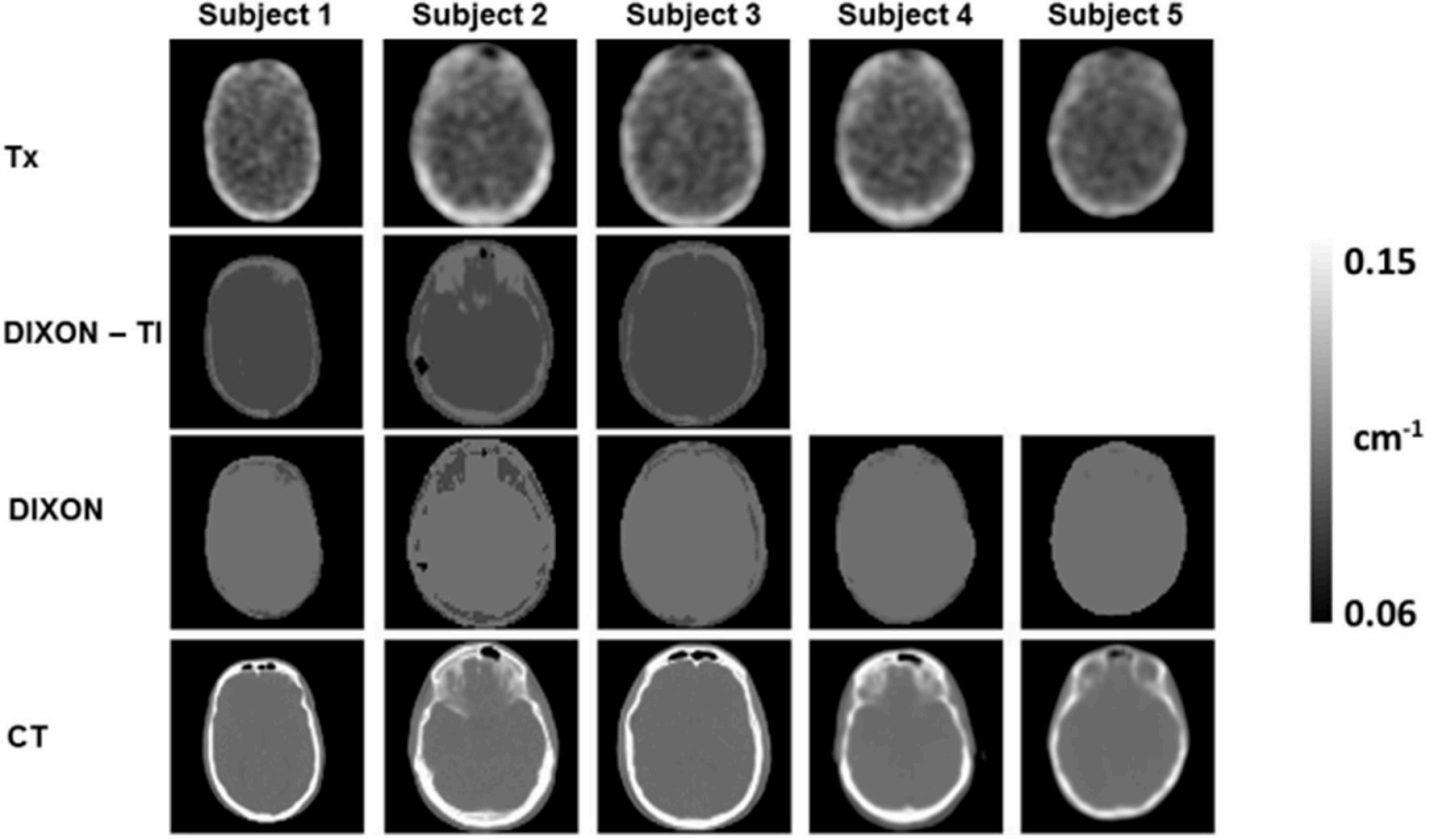
Measured and derived ATN maps obtained for the five subjects. Tissue-inversion (TI) was observed for 3/5 DIXON-ATN maps (row 2). Affected ATN maps were corrected using equation 1, which resulted in new ATN maps as displayed in row 3 (DIXON). A subsequent low-dose CT was obtained for all subjects in a Siemens TPTV system (row 4, CT).

Differences between the two PET-systems (Table 1) were compensated for by the use of resolution-matched reconstruction parameters, through post-filtering of the PET/MRI data and matching of reconstruction parameters in the two PET-systems (van Velden et al., 2009, 2014; Table 2). All datasets were reconstructed using system-matched ATN maps. In the case of PET-only imaging, ATN maps (Figure 1) were obtained from a 5 min pre-injection Tx scan and a daily 90 min blank scan. ATN images were reconstructed and segmented into three tissue classes: bone (linear ATN coefficient 0.125 cm^−^^1^), soft-tissue (0.095 cm^−^^1^), and air (0.0 cm^−^^1^) (Bettinardi et al., 1999). Subsequent ATN maps were forward-projected to calculate AC factors that were used during the reconstruction of the emission data (Table 2).

**Table 1:** Geometrical specifications for the PET-only system (GE Advance) and the PET/MR system (Siemens Biograph mMR).

	**PET-only**	**PET/MR**
PET-detector material	BGO	LSO
Number of detector rings	18	8
Number of imaging planes	35	127
Number of crystals	12,096	28,672
Bore diameter (cm)	60	70
Axial field of view (mm)	152	258

**Table 2:** Reconstruction parameters for the PET data from the PET-only (GE Advance) and PET/MR (Siemens Biograph mMR) system.

	**PET-only**	**PET/MR**
Iterations/subsets	2i 28s	2i 21s
Post-filtering (mm)	6	6
Image matrix	128 × 128 × 35	172 × 172 × 127
Voxel resolution (mm)	3.125 × 3.125 × 4.25	4.173 × 4.173 × 2.03
Attenuation correction	^68^Ga transmission	DIXON-MR and co-registered low-dose CT

*Inter-image-resolution parameters were matched through use of high kernel-width post-filtering of the data (6 mm Gaussian) (van Velden et al., 2009, 2014)*.

In the case of PET/MR, DIXON-ATN maps were acquired using the standard 3-tissue classifications for brain studies: soft-tissue (0.1 cm^−^^1^), fat (0.085 cm^−^^1^), and air (0.0 cm^−^^1^) (Martinez-Moller et al., 2009). Correction of the ATN values was performed according to equation 1 in cases of tissue-inversion of the DIXON-ATN maps (Ladefoged et al., 2014).

The new ATN map was composed of in-phase (in) and opposed-phase (opp) images, based on the ratio of fat (F) and soft-tissue content in each voxel.

(1) $$\begin{array}{l} \;F=0.5^{*}(\text{in}+\text{opp})\\ \;W=0.5^{*}(\text{in}-\text{opp})\\ F_{\text{Norm}}=F/\text{max}(F)\\ W_{\text{Norm}}=W/\text{max}(W)\\ ATN=\{\begin{array}{c} \begin{array}{cc} 0.0854 & F_{Norm}>W_{Norm} \end{array}\\ \begin{array}{cc} 0.1 & W_{Norm}>F_{Norm} \end{array} \end{array} \end{array}$$

Finally, for both PET-only and PET/MR, PET images were reconstructed in two ways: a static image (10–40 min post injection) and a dynamic image sequence (8 × 15 s, 3 × 60 s, 5 × 3 min, 2 × 10 min).

### Image analysis

The T1-weighted MR images and the corresponding PET data were processed with Analyze 8.0 (Biomedical Imaging Resource, Mayo Foundation) and SPM12 (Wellcome Department of Imaging Neuroscience, UCL) software as described previously (Langer et al., 2007). By using the Hammersmith n30r83 3-dimensional maximum probability atlas of the human brain (Hammers et al., 2003) a template of preset volumes of interest (VOIs) was applied to the PET images to extract time-activity curves (TACs) for the following three gray matter regions of interest (ROIs): whole brain (WBGM), insula (INS) and superior parietal lobe (SPL). The WBGM VOI was chosen to estimate the general differences between ATN values and attenuation corrected PET-images. The INS and SPL VOIs were chosen to assess any distance-related bias with regards to the presence and distribution of bone structures (e.g., skull; Samarin et al., 2012). The “bone-distance” effect was assessed through analysis of the relative difference, calculated using equation 2.

(2) $$\%Diff=\frac{SUV_{mMR}-SUV_{Advance}}{SUV_{Advance}}\cdot 100\%$$

Relative differences for the ATN values were calculated to evaluate the regional effects of the three ATN maps used in this study (Tx, DIXON and CT-based). Semi-quantitative standardized uptake value (SUV) images were calculated for the static PET images to obtain relative difference maps between the PET/MR-based and the PET-only reconstructions.

### Kinetic modeling

VOIs were applied to the co-registered dynamic image frame sequences and TACs were extracted as mean activity concentrations (kBq/mL) per reconstructed frame. A standard 1T2K compartment model was fitted to the obtained TACs using PMOD 3.6 (Pmod, Zurich, Switzerland) and the corresponding plasma (corrected for polar radiolabeled metabolites) and whole blood input functions. A time delay of 1 to 5 s was considered in the input function to account for the differences in the time course of radioactivity between the arterial catheter and the arterial capillaries in the brain. The two rate constants describing transfer of radioactivity across the BBB, from plasma to brain (*K*_1_) and from brain to plasma (*k*_2_), were estimated from the data and were used to calculate the distribution volume (*V*_T_ = *K*_1_/k_2_) (Langer et al., 2007).

### Statistical analysis

Differences between imaging parameters obtained using PET/MR and PET-only were assessed using a 2-way analysis of variance (ANOVA) using multiple comparisons. All data was corrected for multiple comparisons using a Holm-Šídák test (Holm, 1979) to improve the power of the tests. A *P*-value < 0.05 was considered statistically significant for all tests.

## Results

### Assessment of ATN maps

Analysis of the DIXON ATN-maps revealed soft-tissue and fat inversion in 3 out of 5 subjects (Figure 1, DIXON-TI). Tissue inversion was corrected using equation 1, and the corrected DIXON-ATN maps were subsequently used for photon AC (Figure 1, DIXON).

**Figure 2:**
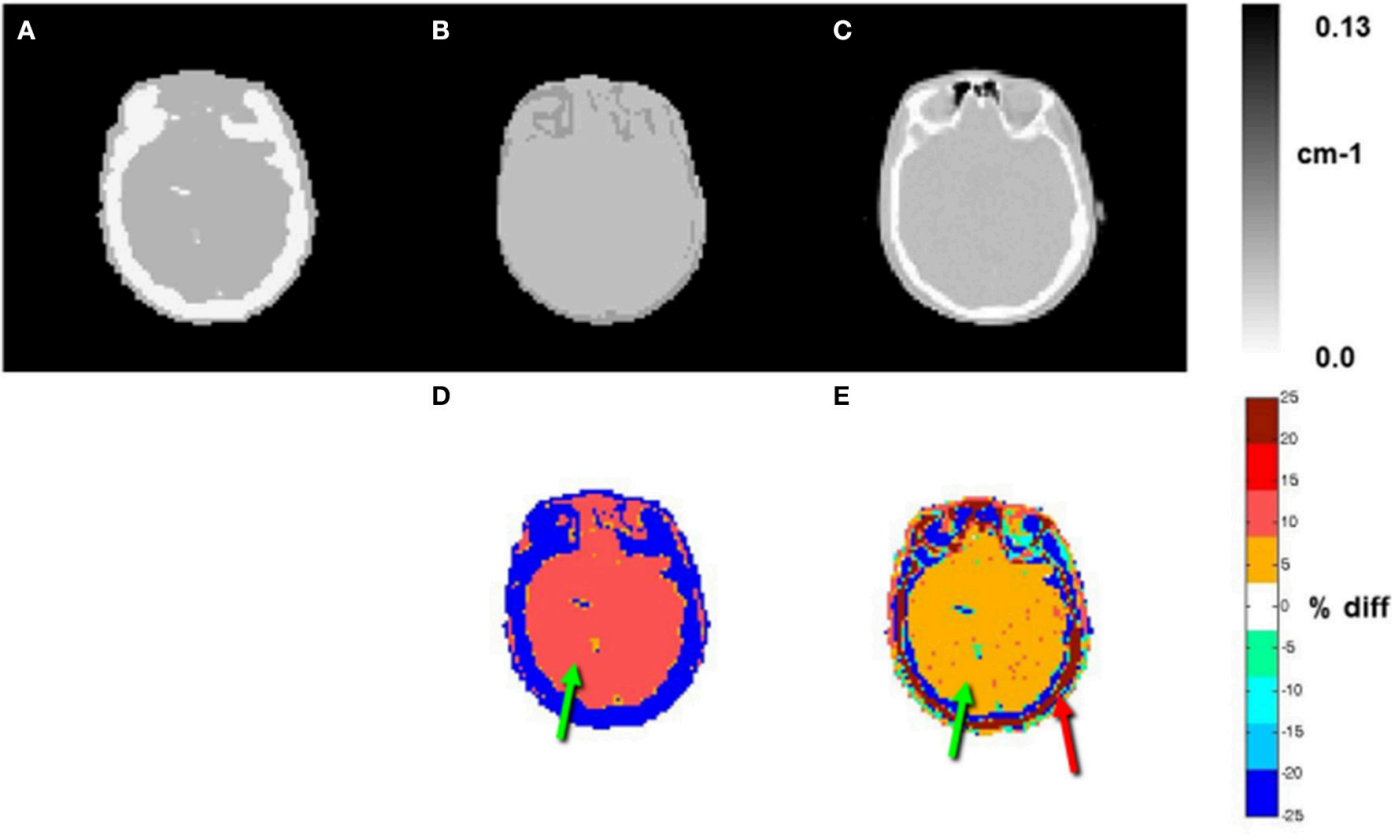
ATN maps of subject 1 (**A**: Tx, **B**: DIXON, and **C**: CT). Relative differences (%) between **B** and **A** and between **C** and **A** are shown in **D** and **E**, respectively. Relative differences for the ATN values for soft tissue of 10 and 5% were observed for the DIXON and CT based ATN maps when compared to the Tx ATN-maps, respectively (green arrows, **D** and **E**). Differences of >25% were observed for the osseous tissues between the CT and Tx-based ATN maps (red arrow, **E**).

Relative difference maps were calculated between the ATN maps employed in the PET/MR and PET-only systems (Figure 2). The relative difference maps revealed systematic offsets in soft-tissue ATN values between the ATN maps used for PET/MR emission data and the Tx-ATN maps, with generally increased ATN values of 10% for DIXON- and 5% for CT-based AC (Figures 2D,E, green arrows). ATN values of bone in the Tx and CT ATN maps differed by more than 25% (Figure 2E, red arrow), at locations where bony structures were present in both ATN maps.

### Assessment of radiotracer concentration

**Figure 3:**
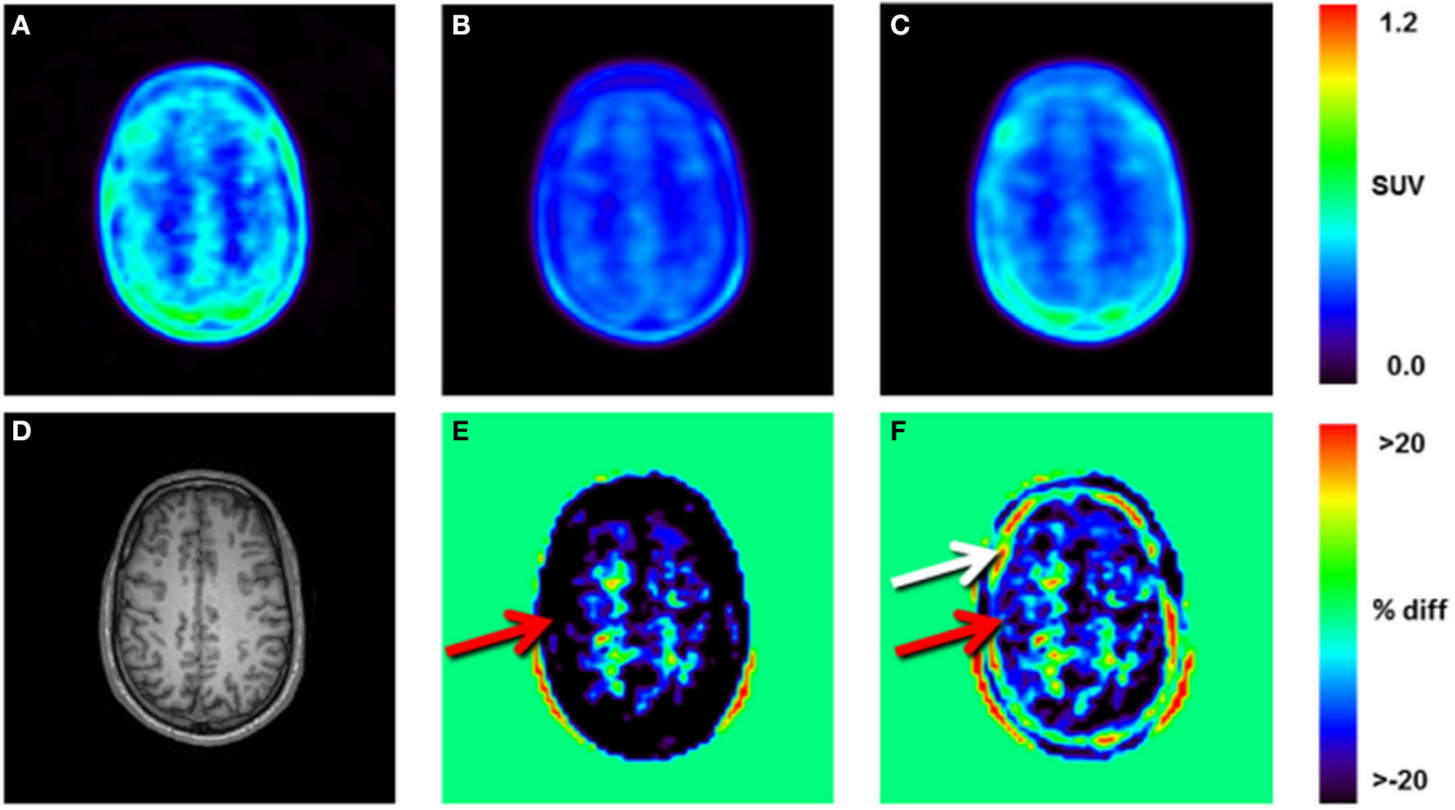
Static reconstructions (10–40 min post injection) of subject 1: **(A)**, PET-only emission data reconstructed using the Tx-ATN map, **(B)** PET/MR emission data reconstructed using the DIXON-ATN map, **(C)** PET/MR emission data reconstructed using the CT-ATN map. **(D)** T1-weighted MR image used for automatic segmentation of the brain-structures, **(E)** relative differences (%) between **B** and **A**, **(F)** relative differences (%) between **C** and **A**. Local differences of up to 55% were observed for areas close to bone, when comparing DIXON- and Tx-reconstructions (red arrow, **E**). Local differences of more than 25% were observed within the bone between CT- and Tx-reconstructions, indicating differences in the assigned bone-ATN values for the two methods (white arrow, **F**).

Radiotracer concentrations expressed as SUVs (10–40 min post injection) derived from the PET/MR system were generally lower than those obtained from the PET-only system, indicating an overall systematic difference in radiotracer concentration between the two systems (Figures 3A–D). Relative difference maps for SUV demonstrate a variable bias dependent on the applied ATN map used to correct the PET/MR emission data (Figures 3E,F). With respect to SUV images created using the DIXON ATN maps, radiotracer concentration at the location of the skull and cortex was underestimated (Figure 3E, red arrow), whereas radiotracer concentration in the white matter (centrum semiovale) was overestimated.

Overestimation of radiotracer concentration in the white matter (centrum semiovale) was also observed for SUV images calculated using the CT-AC, however, with a somewhat lower underestimation of cortical areas (Figure 3F, red arrow) and severe overestimation of radiotracer concentration adjacent to the cortical surface (Figure 3F, white arrow).

Relative to PET-only, the regional radiotracer uptake in PET/MR was underestimated by up to 23% when using DIXON AC and less so (−11%) when using CT AC (Table 3). The average gradient from the outer (cortical) to the central (white matter) brain structures was ~10% in case of the DIXON ATN maps and ~5% for the CT-based ATN maps.

**Table 3:** Average SUV values (mean ± *SD*) obtained from static reconstructions (10–40 min post injection) for whole brain gray matter (WBGM), insula (INS), and superior parietal lobe (SPL).

	**PET-only**	**PET/MR**		**DIXON:Tx**	**CT:Tx**	**DIXON:CT**
	**Tx**	**DIXON**	**CT**	**Relative difference (%)**		
WBGM	0.47 ± 0.03	0.36 ± 0.01	0.42 ± 0.01	−22 ± 10^*^	−10 ± 14	−13 ± 2
INS	0.43 ± 0.03	0.36 ± 0.01	0.39 ± 0.01	−15 ± 14	−7 ± 17	−8 ± 2
SPL	0.47 ± 0.03	0.36 ± 0.01	0.41 ± 0.01	−23 ± 8^*^	−11 ± 9	−13 ± 3

*Relative differences (%) between PET/MR and PET-only based reconstructions reveal a distance-to-bone gradient for the DIXON based reconstruction*.

* *P < 0.05, 2-way ANOVA with Šídák's post-hoc analysis*.

**Figure 4:**
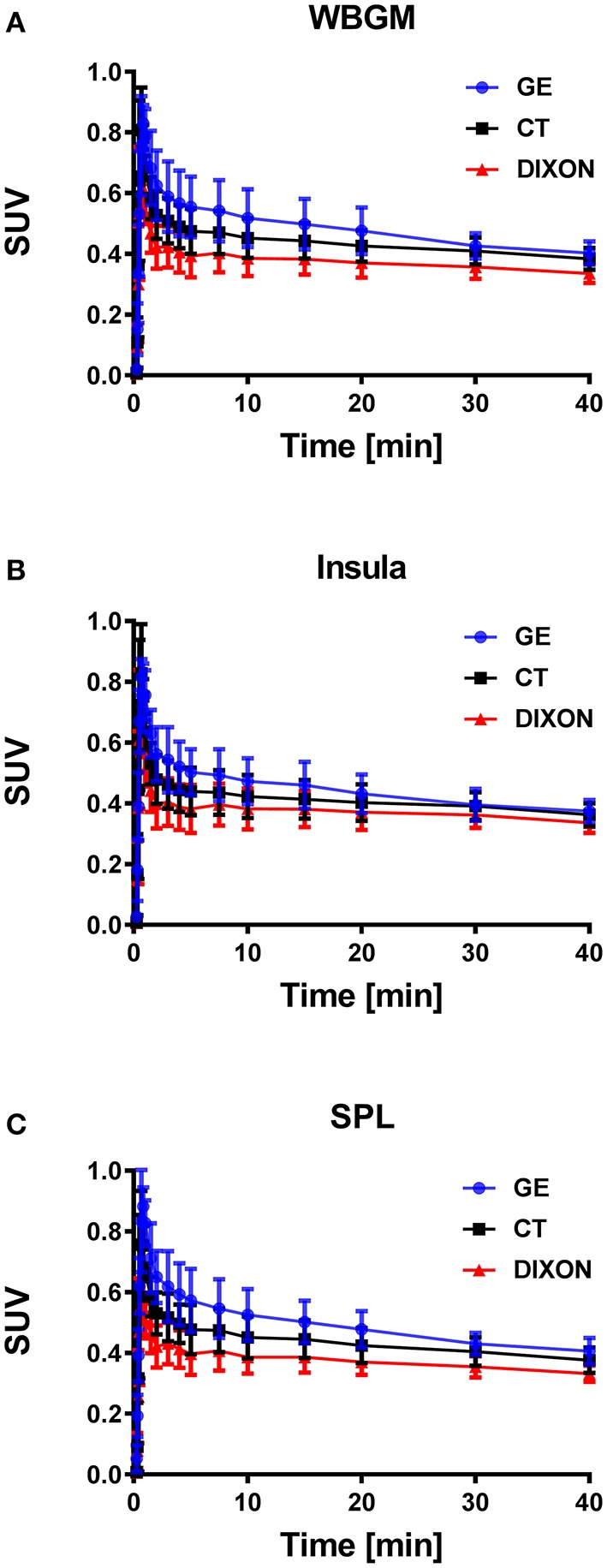
TACs averaged across all five subjects expressed in SUV values for whole brain gray matter (**A**, WBGM), insula (**B**, INS) and the superior parietal lobe (**C**, SPL). Gradient-based differences were observed for the PET/MR based reconstructions, when compared to the PET-only (GE) based reconstruction, with increased differences for the VOIs close to bone.

Figure 4 shows the TACs for the three outlined VOIs (WBGM, INS, SPL). There was a clear rank order with regards to the scale for each region, with TACs derived from the PET/MR system resulting in lower values than those from PET-only.

### Assessment of kinetic parameters

**Figure 5:**
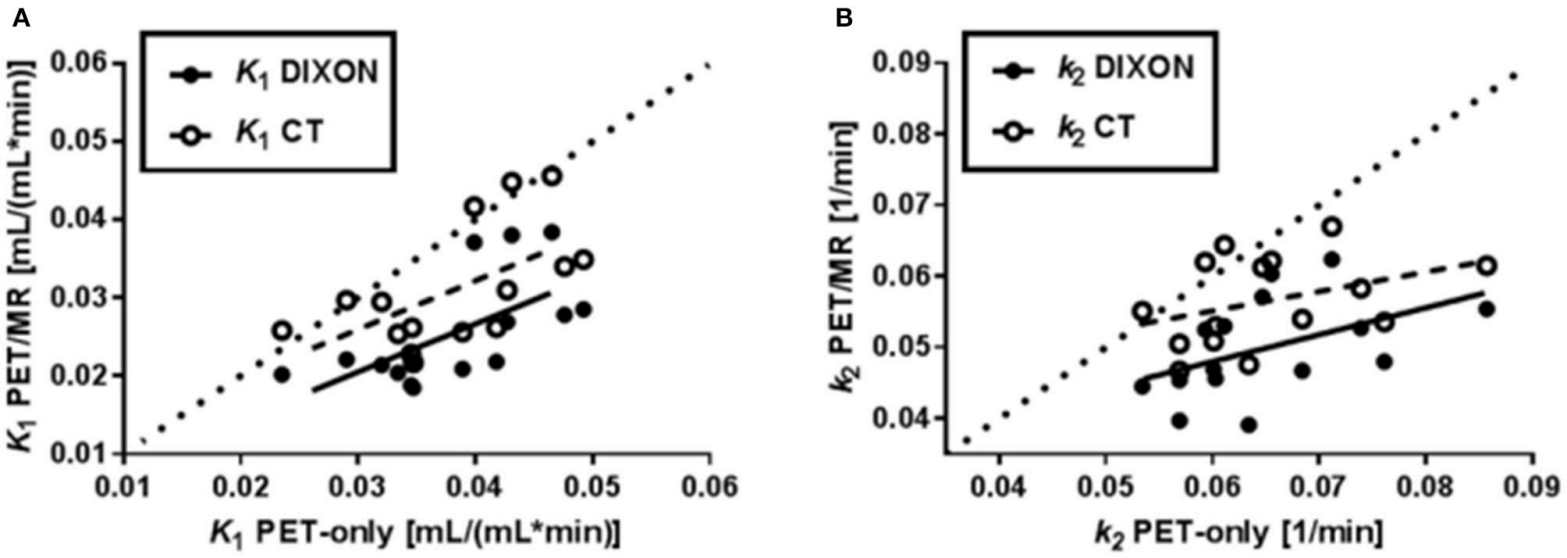
Correlations between kinetic parameters (*K*_1_ and *k*_2_) in the three studied brain VOIs (WBGM, INS, SPL) generated by the PET/MR system (DIXON: filled circles; CT: open circles) and PET-only system. The dotted line in each plot represents identity. In both **A** and **B**, a larger negative bias was seen for the kinetic parameters when DIXON AC was used as compared to CT AC.

Different scaling and shapes of the TACs obtained in the PET-only and PET/MR systems were observed (Figure 4). Consistent with the scale of the TACs, K_1_ values demonstrated a clear negative bias for the PET/MR system derived estimates as compared to the PET-only system (Figure 5). The negative bias for the K_1_ parameter for WBGM was −33% for PET/MR (DIXON) and −16% for PET/MR (CT). Similarly, a negative bias was observed also for k_2_ in WBGM with an average difference of −19% for PET/MR (DIXON) and −9% for PET/MR (CT; Table 4). Overall, a 2-way ANOVA revealed statistically significant differences between the kinetic parameters obtained in the PET/MR and the PET-only systems (*P* < 0.05; Table 4).

**Table 4:** Relative differences for the three VOIs [whole brain gray matter (WBGM), insula (INS), and parietal superior lobe (PSL)] in (%) for the kinetic parameters obtained with the PET/MR system and the PET-only system.

	**WBGM**			**INS**			**SPL**		
	***K*_1_**	***k*_2_**	***V*_*T*_**	***K*_1_**	***k*_2_**	***V*_*T*_**	***K*_1_**	***k*_2_**	***V*_*T*_**
DIXON	−33 ± 14^*^	−19 ± 9^*^	−18 ± 10^*^	−30 ± 18^*^	−27 ± 12^*^	−5 ± 12	−36 ± 12^*^	−23 ± 10^*^	−16 ± 12^*^
CT	−16 ± 18	−9 ± 10	−8 ± 13	−17 ± 22	−15 ± 14^*^	−3 ± 14	−20 ± 15^*^	−13 ± 13	−8 ± 12

*All values are given as mean ± SD*.

* *P < 0.05, 2-way ANOVA with Šídák's post-hoc analysis*.

## Discussion

The main finding of our study is a systematic underestimation of the brain concentration of (R)-[^11^C]verapamil with hybrid PET/MRI when compared to PET-only imaging in the same subjects and following the same protocol. The average magnitude of this underestimation is between −10 and −22% for SUV values and between −9 and −33% for kinetic parameters derived from the arterial blood sample based TACs. Our data indicate that these differences in PET quantification are caused by different methods of AC applied routinely by the two PET systems. As a result, the observed systematic differences in radiotracer concentration preclude the combination of data obtained from the PET-only and PET/MR system without applying higher-order correction schemes for both scale and radiotracer tissue clearance in mono- or multi-centric studies.

It is well*-*known that the quantitative accuracy of different PET systems is affected by differences in system geometries, image reconstruction algorithms as well as in the implementation of AC approaches (Moody et al., 2015; Walker and Sossi, 2015). In addition, there are also differences in instrumentation that affect the obtained emission data (Table 1; Lewellen et al., 1994; Delso et al., 2011). For example, the two PET systems employ different crystal technologies with different scintillation decay times. The shortened scintillation decay time of LSO based PET detectors (~40 ns) in the PET/MR system leads to improved detection rates and thereby to a more accurate estimation of radiotracer distribution in the brain (Pichler et al., 2010; Surti and Karp, 2016). Furthermore, different implementations of iterative reconstruction algorithms substantially add to differences in the quantitative accuracy, preventing a direct comparison of OSEM algorithms used by the two systems. This dependence of image characteristics on different implementations of OSEM reconstruction methods has been previously demonstrated for SPECT studies (Seret and Forthomme, 2009). Although, differences in the implemented reconstruction algorithms affect the overall scale, these inconsistencies are usually without consequences for the reporting medical doctor performing differential diagnoses in clinical SPECT or PET studies.

The ATN maps employed in the PET-only and PET/MR systems make use of photon transmission measurements (Tx and CT) and proton relaxation times (DIXON). Both transmission methods rely on electron density measurements. On the other hand, the DIXON method suffers from tissue-inversion effects due to erroneous fat/water content in the fat/water sequences (Ladefoged et al., 2014). In the current study we observed inversion effects in 60% of the subjects, which exceeds previous reports of 8% for whole body scans (Ladefoged et al., 2014). We corrected the inverted DIXON ATN maps using equation 1 in order to obtain an approximation of the standard DIXON ATN maps. Despite an improved fat/soft-tissue ratio in the corrected ATN maps, an increased fat-segmentation was observed in all ATN-maps (Figure 1).

Detailed inspection of ATN-maps revealed homogeneously elevated soft-tissue ATN values for both DIXON and CT maps when compared to the segmented Tx map (Figure 2). Soft-tissue ATN values were overestimated by ~10% for the DIXON method and by ~5% for the CT method compared to ATN values established by the segmented Tx-map. Although measured AC is considered the gold standard, a segmented Tx-map is likely to yield higher ATN values as a result of suboptimal performance of the segmentation algorithm (Bettinardi et al., 1999). As can be seen in Figure 2, the segmented Tx-maps demonstrated an increased bone-thickness when compared to the CT-ATN maps. Moreover, the conservatively chosen ATN coefficient of 0.125 cm^−^^1^ for bone was underestimated by more than 25% in Tx-based maps (Figure 2E). These differences in the ATN-maps led to quantitative differences in the reconstructed PET-images, however, with reduced magnitude (Figure 3F).

Dissimilarities both in shape and scale were observed for the TACs obtained in the two PET systems, independently of the ATN-maps used during the PET-image reconstruction (Figure 4). The scale of the TACs was found to be lower in case of the PET/MR system as compared to the PET-only system (Table 3, Figure 4). Gradient-based differences between PET/MR and PET-only based reconstructions were observed for all regions, with increased differences in areas close to bone structures, confirming previous studies that evaluated differences between DIXON and CT-based ATN methods (Andersen et al., 2014). We determined a negative bias with respect to the *K*_1_ parameter obtained from the PET/MR system in consistency with the lower scale of the TACs relative to the sampled arterial input function. This negative bias in *K*_1_ was significantly higher for the DIXON-ATN derived TACs than for the CT-ATN derived TACs (−33 ± 14% vs. −16 ± 18%, *P* < 0.05; Figure 5). Moreover, a negative bias was also observed for the k_2_ values derived from PET/MR when compared to the PET-only reconstructions. Differences were −19 ± 9% for DIXON and −9 ± 10% for CT (Table 4, Figure 5).

The differences in *k*_2_ were caused by an apparently slower washout of radiotracer from 15 min onwards (Figure 4). These findings strongly suggest that pooling of quantitative parameters derived from the two PET systems within a single study should be avoided. Further, these results support existing knowledge that limitations of DIXON based ATN method may not only affect intra-scan quantification, but also inter-system quantification. However, the use of the DIXON ATN maps still suffice in clinical assessment of brain lesions further away from bone, if absolute quantification is not needed (Rausch et al., 2017). This insight may help other centers facing the same tasks of transferring imaging protocols from PET-only to PET/CT or PET/MR systems, given the upcoming end-of-life-cycle for the PET-only systems. In the current study we used the standard vendor provided DIXON ATN maps, although experimental new and improved AC methods have been recently suggested [e.g., Ladefoged et al., 2017]. Many of the new MR-AC methods provide quantitative accuracy within a ±5% variation of the results obtained through CT-based AC (Ladefoged et al., 2017). The use of these methods would, however, not facilitate quantitative reproducibility in the two PET-systems, as differences of more than 15% were observed for quantitative values. Furthermore, is DIXON AC, or variants thereof, still the main implemented AC method on commercially available PET/MR systems, despite its known drawbacks (Wagenknecht et al., 2013; Koesters et al., 2016).

The limitations of this study include the small number of subjects and the homogeneous brain uptake of the chosen radiotracer. Although, our subject cohort size has a negative effect on statistical power, involvement of a larger number of subjects was prohibitive due to the complexity of the study design that included the logistics of radiotracer delivery as well as data acquisition on three different PET systems on a single day. Furthermore, while uptake of (R)-[^11^C]verapamil in brain can be modeled with a well-identifiable 2 parameter model, the relative homogenous tracer distribution renders a comparison with radiotracers that are characterized by distinct focal uptake a challenge. Consequently, further studies using a variety of radiotracers with diverse brain uptake patterns are necessary in order to complement as well as extend the applicability of our findings.

## Conclusion

Systematic differences in the magnitude of radiotracer concentration, introduced by differences in AC maps, were observed between a PET/MR and PET-only system. Given the observed systematic differences of up to −22% in scaling of the TACs obtained for the PET/MR and the PET-only system, a combination of quantitative data derived from the two PET systems is not permissible without proper scaling adjustments.

## Author contributions

Designing work: ML, OM, TB, OL, and MB; Data acquisition and analysis: ML, OM, TB, IR, OL, and MB; Data interpretation, drafting of the work, final approval and agreement: All of the authors. Revision of the paper: ML, TB, and MB.

### Conflict of interest statement

The authors declare that the research was conducted in the absence of any commercial or financial relationships that could be construed as a potential conflict of interest.
